# The Opportunities and Challenges of Peroxisome Proliferator-Activated Receptors Ligands in Clinical Drug Discovery and Development

**DOI:** 10.3390/ijms19082189

**Published:** 2018-07-27

**Authors:** Fan Hong, Pengfei Xu, Yonggong Zhai

**Affiliations:** 1 Beijing Key Laboratory of Gene Resource and Molecular Development, College of Life Sciences, Beijing Normal University, Beijing 100875, China; hongfanky@126.com; 2 Key Laboratory for Cell Proliferation and Regulation Biology of State Education Ministry, College of Life Sciences, Beijing Normal University, Beijing 100875, China

**Keywords:** PPAR, ligand, T2DM, dyslipidemia, TZDs

## Abstract

Peroxisome proliferator-activated receptors (PPARs) are a well-known pharmacological target for the treatment of multiple diseases, including diabetes mellitus, dyslipidemia, cardiovascular diseases and even primary biliary cholangitis, gout, cancer, Alzheimer’s disease and ulcerative colitis. The three PPAR isoforms (α, β/δ and γ) have emerged as integrators of glucose and lipid metabolic signaling networks. Typically, PPARα is activated by fibrates, which are commonly used therapeutic agents in the treatment of dyslipidemia. The pharmacological activators of PPARγ include thiazolidinediones (TZDs), which are insulin sensitizers used in the treatment of type 2 diabetes mellitus (T2DM), despite some drawbacks. In this review, we summarize 84 types of PPAR synthetic ligands introduced to date for the treatment of metabolic and other diseases and provide a comprehensive analysis of the current applications and problems of these ligands in clinical drug discovery and development.

## 1. Introduction

**Figure 1:**
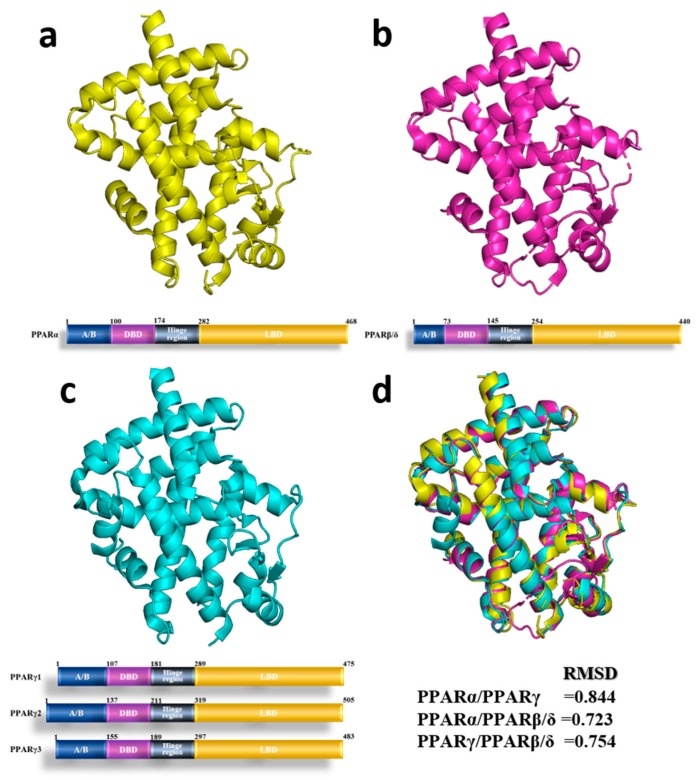
3D structure and schematic structure of human Peroxisome proliferator-activated receptors (PPARs). 3D structure and schematic structure of PPARα (1I7G [16]) (**a**) PPARβ/δ (1GWX [17]) (**b**) and PPARγ (1FM6 [18]) (**c**,**d**) 3D structure superposition of PPARα (yellow), PPARβ/δ (magenta) and PPARγ (cyan) and RMSD value of three PPARs within pairwise comparison.

Peroxisome proliferator-activated receptors (PPARs) are a group of nuclear receptors (NRs) that play essential roles in the regulation of several physiological processes, including cellular differentiation and development, whole-body energy homeostasis (carbohydrate, lipid, protein) and tumorigenesis [1]. PPARs are ligand-activated transcription factors and consist of a DNA binding domain in the N-terminus and a ligand binding domain (LBD) in the C-terminus [2,3]. The family of PPARs comprises three isoforms: PPARα (NR1C1), PPARβ/δ (NR1C2) and PPARγ (NR1C3) [2] and their 3D structures are shown in Figure 1. PPARα is highly expressed in metabolically active tissues and PPARγ which has three forms: PPARγ1, PPARγ2 and PPARγ3 is mainly expressed in white and brown adipose tissue [4]. The least known isoform is PPARβ/δ, which is expressed ubiquitously in virtually all tissues. After interaction with agonists, PPARs are translocated to the nucleus, where they heterodimerize with the retinoid X receptor (RXR) [5]. Then, PPAR-PXR binds to peroxisome proliferator hormone response elements (PPREs) [2] and regulates target genes. All three PPARs have natural agonists, namely, a variety of polyunsaturated long-chain fatty acids and arachidonic acid derivatives.

PPARs regulate genes that are important in cell differentiation and various metabolic processes, especially lipid and glucose metabolism. In both rodents and humans, PPARs are genetic sensors for lipids and modulate genes through the promotion of reverse cholesterol transport, reduction of total triglycerides (TGs) and regulation of apolipoproteins, thermogenesis and glucose metabolism. PPARα regulates the catabolism of fatty acids in the liver by inducing the expression of fatty acid transport protein (FATP) [6], FAT [7], long-chain fatty acid acetyl-CoA synthase (ACS) [8], enoyl-CoA hydratase/dehydrogenase multifunctional enzyme [9] and keto-acyl-CoA thiolase [10] enzymes. PPARγ influences the storage of fatty acids in adipose tissue by regulating the expression of numerous genes, including AP2 [11], PEPCK [12], acyl-CoA synthase [13] and LPL [14]. Furthermore, PPARβ/δ activation also improves lipid homeostasis, prevents weight gain and increases insulin sensitivity [15]. Accordingly, PPARs are considered important targets for the treatment of metabolic syndrome and choreographers of metabolic gene transcription.

PPARs are also called lipid and insulin sensors [2]. Hence, many synthetic agonists of PPARs have different properties and specificities, having been developed for the treatment of different clinical outcomes over the past several decades [19,20,21]. For example, PPARα activators such as fibrates (fenofibrate, clofibrate) are useful drugs for the treatment of dyslipidemia. They increase HDL, decrease TG and have no effects on low-density lipoprotein (LDL). PPARγ is a target of synthetic insulin sensitizers thiazolidinediones (TZDs), including pioglitazone and rosiglitazone, which were used in the treatment of type 2 diabetes mellitus (T2DM). Dual agonists of PPARα/γ, such as glitazar, have been developed and have recently become available for the combined treatment of T2DM and dyslipidemia. Of course, there are many drugs targeting PPARs for the clinical treatment of various diseases. However, many drugs have been limited or terminated in the clinical stage by their side effect profiles. TZDs are well known to have prompted an alert by the US Food and Drug Administration (FDA) due to adverse effects, such as fluid retention, congestive heart failure (CHF) and adipogenic weight gain [22]. In this review, we summarize the use of some PPAR agonists in therapeutic treatment, with a focus on both the pros and the cons of PPARs as key regulators of glucose and lipid metabolism. Thus far, current clinical data exists for the use of 84 PPAR ligands for the treatment of diabetes mellitus, lipid metabolism disorder and other diseases (Table 1).

## 2. PPAR Ligand Therapeutics in Diabetes Mellitus

Diabetes mellitus is a chronic, “whole-life“ condition that increases the body’s blood glucose levels. There are three main types of diabetes: type 1 diabetes (insulin dependent), T2DM (insulin resistance or insulin insensitivity) and gestational diabetes [23]. Diabetes mellitus and diabetic complications constitute the most important economic cost of the disease and represent a significant economic burden for the healthcare systems of developed countries [24]. As important modulators of lipid and glucose metabolism, PPAR ligands were used to treat T2DM and diabetes-associated complications.

### 2.1. Type 2 Diabetes

The majority of patients with diabetes are diagnosed with T2DM, which affects at least 250 million people worldwide [25]. Insulin resistance is a major determinant of T2DM, which involves some defects of response to pancreatic insulin in muscle and liver cell [26]. The main treatment for diabetes mellitus is to lower the blood glucose levels to reach as close to normal as possible. Many pharmacological agents are utilized in patients with type 2 diabetes, such as TZDs, biguanide, GLP-1 agonists, DPP-4 inhibitors and SGLT2s. Here, we summarize the market value of the ligands of PPAR-treated type 2 diabetes (Table 2).

TZDs, as PPARγ agonists, are increasingly being used to counteract the effects of diabetes by regulating the transcription of insulin-responsive genes, thereby enhancing insulin sensitivity in adipose tissue, skeletal muscle and liver to help reduce plasma glucose and insulin [26]. TZDs were developed in the late 1990s and have been used to treat up to 26% of people with diabetes mellitus [27]. In the market, the main approved TZD drugs for the treatment of type 2 diabetes are rosiglitazone, pioglitazone, lobeglitazone sulfate and these drugs often used combination with metformin or other antidiabetic drugs. Rosiglitazone (Rosiglitazone Maleate) is a pure ligand of PPARγ without PPARα-binding action [28]. The pharmaceutical company GlaxoSmithKline (Brentford, UK) marketed it as Avandia^®^, a standalone preparation and combined it with metformin as a compound (Avandamet). Another combination drug approved by the FDA is Avandaryl^®^ (with glimepiride) [29]. Studies on animal models of insulin resistance and diabetes have shown that rosiglitazone prevents the onset of hyperglycemia, proteinuria and pancreatic islet cell degeneration [23]. In patients with T2DM, rosiglitazone reduces fasting plasma glucose (FPG), HbA1c, insulin, C-peptide and postprandial serum glucose [30]. However, in rosiglitazone monotherapy, clinically significant side effects such as edema, anemia and weight gain are frequently reported with a conventional dosage of drug [31]. Moreover, patients with unstable heart failure (HF) and patients with a history of myocardial infarction (MI) should avoid the use of rosiglitazone due to the increased risk of cardiovascular disease (CVD) [32]. Pioglitazone hydrochloride is the hydrochloride salt of thiazolidinedione with antidiabetic properties and potential antineoplastic activity [33]. Pioglitazone monotherapy significantly improves HbA1c and FPG while producing beneficial effects on serum lipids in patients with type 2 diabetes with no evidence of drug-induced hepatotoxicity [34]. However, raising the dose and time of pioglitazone use increased the risk of bladder cancer and reached statistical significance after 24 months of exposure. Thus, the FDA issued an alert about a potential relation between the incidence of bladder cancer and the prescription of pioglitazone [35]. However, a recent meta-analysis based on 193,099 persons in the bladder cancer cohort conservatively suggested that pioglitazone use was not associated with a statistically significant increased risk of bladder cancer [36]. Given the many adverse effects of troglitazone, combination therapy can better treat type 2 diabetes. Alogliptin benzoate, a dipeptidyl peptidase-4 inhibitor, has a blood glucose-dependent insulinotropic effect via elevated concentrations of glucagon-like peptide-1 [37,38]. Pioglitazone/alogliptin combination therapy was effective and generally well tolerated in Japanese subjects with T2DM and is considered to be useful in clinical settings [39]. 

Lobeglitazone sulfate, a novel PPARγ agonist, was conceptually designed by modification of the rosiglitazone structure with a substituted pyrimidine [40]. Lobeglitazone has a *p*-methoxyphenoxy group at the 4-position of the pyrimidine moiety [41] and is structurally similar to two well-known TZD drugs, rosiglitazone and pioglitazone. These substituted pyrimidines were selected based on their empirical effects on triglyceride accumulation in adipocytes in vitro and their glucose-lowering and lipid-modulating activities in diabetic mice in vivo [42,43]. In contrast to other TZDs, lobeglitazone is mainly excreted in the feces, reducing the concerns about the risk of bladder cancer in the mice [44] and rats [45]. In the study of lobeglitazone in patients with T2DM, lobeglitazone showed a favorable balance of efficacy and safety during the extension study [46]. In pharmacokinetic studies in healthy adults, lobeglitazone was well tolerated and did not significantly affect the pharmacokinetics of metformin or vice versa [47]. In addition, the glucose-lowering effect of lobeglitazone is more promising in obese patients with inadequate glycemic control, long-term diabetes and severe insulin resistance.

The full activation of PPARγ is related to the phosphorylation of PPARγ Ser273, which results in a series of side effects [48]. Therefore, many new insulin sensitizers based on the pharmacology of the TZDs for clinical use have focused on the selective activation of PPARs in the clinical stage. Here, we summarize the PPAR ligands used to treat type 2 diabetes in the clinical stage (Table 3).

Chiglitazar is a configuration-restricted non-TZD PPAR pan agonist with AC50 values of 1.2, 0.08 and 1.7 μM in CV-1 cells for PPARα, PPARγ and PPARδ, respectively and is currently in phase III clinical development in China [49]. In animal studies, chiglitazar demonstrated comparable antidiabetic effects to those of rosiglitazone but had fewer adverse effects involving body weight and fat pad weight increases in KKAy and db/db diabetic mouse models. Clinical studies (phase IIa and IIb) also show that the complete dose range of chiglitazar has a well-tolerated safety profile in patients with T2DM [49]. Its overall encouraging profile in terms of efficacy versus toxicity might be related to the balanced activity of chiglitazar towards different PPAR subtypes [49]. KDT-501 is a compound chemically derived from hops that has antidiabetic effects in rodents [50]. Multiplex analysis of gene expression revealed that KDT-501 enhanced the expression of PGC1α and PPARα but showed no evidence of activating PPARγ [51]. The oral administration of KDT-501 in DIO mouse and ZDF rat models of diabetes reduced plasma HbA1c and improved glucose metabolism. A recent study showed that KDT-501 treatment reduced plasma triglyceride levels in an open-label, phase II clinical trial including nine obese, insulin-resistant subjects [52]. Plasma total and high-molecular-weight (HMW) adiponectin were higher and plasma tumor necrosis factor alpha (TNFα) also reduced after KDT-501 treatment [52].

Many other drugs are currently in clinical studies, including naveglitazar (phase II, Lilly (Indianapolis, IN, USA)), AVE-0897 (phase І, Genfit (Originator) Sanofi) and ZY-H2 (phase І, Zydus Cadila (Ahmedabad, Gujarat, India)).

Due to safety and tolerability issues such as weight gain, edema, CHF and bone fracture, many drugs have been terminated during the clinical research stage. For example, a class of pharmaceutical molecules exhibiting PPARα/γ dual effects is known as the “glitazars,” including aleglitazar, ragaglitazar, tesaglitazar, sipoglitazar, muraglitazar, cevoglitazar and naveglitazar [53]. They have been investigated for potential use in treating T2DM and dyslipidemia simultaneously. Here, we summarize the “glitazar” drugs for the treatment of T2DM that were terminated in the clinical research stage (Table 4).

The dual PPARα/γ agonist alegitazar exerts antihyperglycemic and lipid profile-modifying effects [54], leading to insulin-sensitizing and glucose-lowering activities and favorable effects on lipid profiles and biomarkers of cardiovascular risk [55]. However, the development of aleglitazar was halted because of a lack of cardiovascular efficacy and PPAR-related side effects in patients with T2DM post-acute coronary syndrome [56]. Ragaglitazar was mentioned as carcinogenic to the urinary bladder in Sprague-Dawley male rats exposed to 50 mg/kg/day (approximately 10 times the human exposure) in a 2-year carcinogenicity study [57]. Ragaglitazar was in phase III trials by Novo Nordisk (Copenhagen, Denmark) but was terminated in July 2002 because it caused urinary bladder tumors in mice [58]. Similarly, the development of tesaglitazar was discontinued because it severely increased serum creatinine in diabetic patients. Sipoglitazar, an azolealkanoic acid derivative, exhibits selective PPAR agonist activities towards PPARs. For example, sipoglitazar was used to treat patients with metabolic syndrome and T2DM through improving peripheral insulin sensitivity, lowering the lipid content of bodies and reducing body weight [59]. Sipoglitazar reached phase II clinical trials by Takeda for the treatment of diabetes; however, this research has been discontinued. The development of reglitazar, a PPARγ agonist that is structurally similar to TZDs and exhibits some degree of PPARα activity, was discontinued due to its lower than expected efficacy after phase II clinical trials [60].

In brief, no “glitazar” drugs, which also include muraglitazar (NDA Filing US, Bristol-Myers Squibb (Ney York, NY, USA), imiglitazar (phase Ш, Takeda (Tokyo, Japan)), indeglitazar (phase II, Pfizer (Ney York, NY, USA)), farglitazar (phase II, GlaxoSmithKline) and peliglitazar (phase II, Bristol-Myers Squibb (Ney York, NY, USA)), has ever been approved for clinical use due primarily to the concern of cancer risk in animals, despite their promising effects on related metabolism.

In addition to “glitazar,” other PPAR agonists for the treatment of T2DM have also halted development in the clinical research stage are lost development, as shown in Table 5.

Balaglitazone is a novel partial agonist of PPARγ that was developed by Dr. Reddy’s laboratories in India. As a selective partial PPARγ agonist, balaglitazone presents a better safety profile than full agonists and cuts down HbA1c levels significantly. Balaglitazone provides robust glycemic control as an add-on to insulin therapy and a trend towards less severe side effects was observed in phase Ш trials [61]. However, the investment was halted in 2011. FK-614, a novel non-TZD PPARγ agonist, was as an antidiabetic agent and displays beneficial effect on improving insulin resistance [62]. FK-614 induces adipocyte differentiation by stimulating PPARγ in Zucker obese rats and altering WAT characteristics and improving systemic insulin sensitivity [63,64]. However, Astellas (Tokyo, Japan) (pharmaceutical company developing FK-614) has discontinued the development of FK-614 for the treatment of type 2 diabetes because its efficacy and safety parameters showed insufficient advantages over competitors [65]. Ciglitazone improves glycemic control by increasing insulin sensitivity [66]. Long-term use of ciglitazone treatment can significantly reduce blood glucose in diabetic db/db mice, accompanied by recovery of glomerular immunopathology and renal tubular disorders [67]. Ciglitazone had been in phase II clinical trials by Takeda for the treatment of diabetes mellitus. However, this research has been discontinued [58]. In addition, many drugs are lost from development in the clinical stage, including rivoglitazone hydrochloride (phase Ш, Daiichi Sankyo (Tokyo, Japan)), ONO 5129 (phase II, Ono), EML-4156 (phase II, Merck Serono), netoglitazone; isoglitazone (phase II, Mitsubishi Tanabe Pharma (Originator) Perlegen Sciences), PN-2034 (phase II, Wellstat (Originator) Sanofi), Edaglitazone (phase II, Roche (Basel, Switzerland)), darglitazone sodium (phase І, Pfizer), AVE-5376 (phase І, Sanofi), DS-6930 (phase І, Daiichi Sankyo) and E-3030 (phase І, Eisai).

As mentioned above, in many clinical studies of TZDs targeting PPARγ have encountered problems with the adverse effects of TZDs and the use of these drugs has been limited, or they have been withdrawn from the markets in the United States, Europe and other countries [68]. However, the debate on the safety of TZDs continues and some scientists are also attempting to develop new classes of insulin sensitizers. Thus, there is still a need for novel TZDs. The selective modulation of PPARγ provides the opportunity to improve the safety profile while retaining the desirable therapeutic effects.

### 2.2. Diabetes-Associated Complications

Diabetes increases the risk of cardiovascular disease [25], retinopathy [69], renal failure [70] and peripheral vascular disease. Moreover, diabetes-associated complications and comorbidities also add to the lethality of T2DM [71]. Similarly, PPAR agonists have a good therapeutic effect on diabetes-associated complications, such as diabetic dyslipidemia, hypertension and Alzheimer’s disease. Here, we summarize the PPAR ligands used to treat diabetes-associated, as shown in Table 6.

A very common metabolic abnormality associated with diabetes is dyslipidemia, which occurs in over 50% of T2DM patients and is often unresponsive to statin treatment [72,73]. Saroglitazar, a novel glitazar compound, is indicated mainly for T2DM patients for the treatment of diabetic dyslipidemia and hypertriglyceridemia not controlled by statin therapy [74]. Saroglitazar has shown dual PPARα/γ agonism with a predominant PPARα and moderate PPARγ activity has shown encouraging results at all stages of clinical trials. So far, Saroglitazar has been unrelated to any serious adverse events and it has not any adverse effects of weight gain and edema associated with TZDs [74]. Another drug used to improve dyslipidemia is HPP593, an effective selective PPAR δ agonist with no off-target activity. HPP593 exhibits an anti-diabetic effect in animal models of T2DM and also has demonstrated a reduction in LDL cholesterol and TGs and improved HDL cholesterol content. HPP593 is now in phase I clinical trials by High Point Pharmaceuticals (a subsidiary of vTv Therapeutics) for the treatment of diabetes and dyslipidemia [58]. K-111 is a new insulin-sensitizer with PPARα activity but without PPARγ activity [75]. K-111 is structurally unrelated to thiazolidinediones; however, it has been shown to exert antihyperinsulinemic and lipid-lowering activity in rodents [75]. Furthermore, K-111 exhibits various pharmacological therapies for insulin sensitivity [76], dyslipidemia [77] and hypertension [78] in a nonhuman primate model. CLX-0921 is a weak activator of PPAR but retains effective glucose uptake activity in vitro and has equivalent glucose lowering activity in vivo to rosiglitazone. In addition, compared to rosiglitazone, CLX-0921 showed a 10-fold reduction in vitro adipogenic potential and increased glycogen synthesis, which is usually independent of rosiglitazone or pioglitazone [79]. In addition to treatment with diabetes, CLX-0921 has shown an inhibitory effect on lipopolysaccharides-induced TNFα production in human monocytes. Mechanistic studies showed that some of the effects of CLX-0921 are attributable to the inhibition of IκB phosphorylation and subsequent inhibition of NFκB activation, an effect not seen for other thiazolidinediones [80].

Among the patients with T2DM, approximately 10% developed diabetic nephropathy (DN) [81]. DN remains the leading cause of end-stage renal disease (ESRD) in the United States [82]. In the process of diabetic glomerular damage, podocytopathy is extremely important [83]. PPARγ is located in all three types of glomerular cells with prominent expression in podocytes [84]. The endogenous lipid electrophile 10-nitrooctadec-9-enoic acid (nitro-oleic acid, NO2-OA) can target and activate PPARγ. In animal models, NO2-OA has demonstrated benefits in a variety of metabolic and circulatory diseases, including hypertension [85] vascular neointimal proliferation [86], obesity with metabolic syndrome [87] and hyperglycemia in diabetes [88]. NO2-OA improved renal ischemia-reperfusion injury by inhibiting Bax translocation and activation and the subsequent mitochondria-dependent apoptotic cascade by regulating PPAR [89]. AMG-131, a novel, non-TZD, selective PPARγ modulator, is under development by InteKrin Therapeutics, Inc. for the treatment of T2DM and multiple sclerosis (MS). AMG-131 displays robust glucose-lowering activity in rodent models of diabetes while exhibiting a reduced side effect profile compared to marketed TZDs [90]. In phase I and II clinical trials, AMG-131 was well tolerated, without any serious adverse events or reports of fluid retention [91]. In addition, SAR-351034 is also a PPAR agonist intended for the treatment of diabetes and dyslipidemia.

Numerous dual PPAR agonists have been developed; however, because of collateral side effects, none of these agents apart from saroglitazar has been marketed. Here, we summarized the PPAR drugs for the treatment of diabetes-associated complications that were terminated in the clinical research stage (Table 7).

The sulfur-substituted fatty acid analog tetradecylthioacetic acid (TTA) is a pan–PPAR activator that reduces plasma lipids and enhances hepatic fatty acid oxidation in rodents [92]. In rats, TTA causes a significant reduction in plasma triacylglycerol accompanied by increased mitochondrial and peroxisomal β-oxidation in the liver [93,94]. TTA might exert beneficial effects by increasing complete fatty acid oxidation and TAG formation, thereby improving overall energy metabolism and fatty acid handling in T2DM skeletal muscle [95]. However, the development of TTA has been discontinued due to deleterious effects on the heart, including reduced cardiac efficiency, impaired mitochondrial respiratory capacity and reduced functional recovery following ischemia-reperfusion [96]. Cevoglitazar, a dual agonist of PPARα/γ, is currently being developed for the treatment of dyslipidemia and obesity associated with T2DM [97]. Cevoglitazar has demonstrated both antiobesity and antidiabetic properties in mice and monkey models of obesity, providing a potential novel approach for the treatment of human obesity, diabetes and related metabolic disorders by using a single small molecule [98]. In phase I trials, the compound was reportedly more efficacious than fenofibrate in lowering lipids and at last report, it was also in phase IIa trials for the treatment of dyslipidemia [99]. However, Novartis (Basel, Swiss) announced that they had terminated the development of cevoglitazar without providing a reason [99]. The dual PPARα/γ ligand MK-0767, also known as KRP-297, was found to have potent insulin-sensitizing and antihyperglycemic activities in a preclinical model of obese T2DM, ob/ob mice [100,101]. The effects of the compound on triglyceride and cholesterol levels were assessed in hamster and dog, two species that have previously provided predictive data on the beneficial actions of other drugs, such as fibric acid derivatives and statins, currently used to treat human dyslipidemia [102]. However, MK-0767 has been noted to produce urothelial cancer and hemangiosarcoma in rodents and thus, its development has been discontinued [103]. Sodelglitazar is a panagonist active towards all three PPARs. Sodelglitazar reached phase II clinical development for the treatment of T2DM and metabolic syndrome [104]. However, this research has been discontinued because of serious safety concerns [105]. DSP-8658 is a nonthiazolidinedione compound that markedly improves glucose metabolism and increases β-cell volume, reduces adipocyte size and ameliorates plasma TG levels in diabetic mice [106]. DSP-8658 reached phase I clinical trials by Dainippon Sumitomo for the treatment of Alzheimer’s disease and type 2 diabetes. However, this research has been discontinued [58].

In addition, many drugs intended for the treatment of diabetes—associated complications have been terminated at the clinical research stage, including AVE-0847 (phase II, Sanofi), KRP-101 (phase II, Kyorin), ARH-049020 (phase I, AstraZeneca), LY-510929 (phase I, Lilly) and GSK-376501 (phase I, GlaxoSmithKline).

## 3. PPAR Ligand Therapeutics in Lipid Metabolism Disorder

The PPAR family of NRs is implicated in the regulation of lipid homeostasis and represents a valuable therapeutic target for obesity. Obesity, defined as a body mass index (BMI) ≥ 30 kg/m^2^, is an international public health issue that affects the quality of life, increases the risk of illness and raises healthcare costs in countries in all parts of the world [107,108,109]. Obesity is strongly associated with insulin resistance [110], nonalcoholic fatty liver disease (NAFLD)/nonalcoholic steatohepatitis [111], dyslipidemia [112] and atherosclerosis [113]. In this metabolic derangement, PPARα agonists, mainly fibrates and omega-3 fatty acids, act as powerful TG-lowering agents. They are used mainly to treat metabolic dyslipidemia [21], which is an abnormal amount of lipids including triglycerides, cholesterol and fat phospholipids in the blood.

### 3.1. Dyslipidemia

Hyperlipidemia, the most common type of dyslipidemia, is a condition of elevated lipid levels and is known to accelerate the process of atherosclerosis, which may prove fatal in the development of various cardiovascular diseases. Increases in lipids, such as LDL, cholesterol and triglycerides, are mainly responsible for hyperlipidemia. The current pharmacotherapy for hyperlipidemia includes statins, niacin, fibric acid derivatives and cholesterol absorption inhibitors [114]. Fibrates, such as PPARα activators, have been used for decades in the management of combined dyslipidemia [115]. Fibrates can lower triglyceride levels by an average of 36% and raise levels of small HDL particles [116]. Fibrates increase the production of apolipoprotein AI (apoAI) and AII in the liver, which in turn stimulates HDL production. Triglyceride synthesis is also decreased and lipoprotein lipase activated in response to treatment with fibrates, reducing VLDL synthesis and enhancing its clearance [117]. In addition to fibrates, these approved drugs improve lipid metabolism, as shown in patients with dyslipidemia treated with bezafibrate [118], fenofibrate [119] and ciprofibrate [120] and to a lesser extent in patients treated with gemfibrozil [121]. The approved PPAR ligand drugs for the treatment of dyslipidemia are shown in Table 8.

Clofibrate, the fibric acid derivative, was first approved for use in the United States in 1967 and was the most universally used lipid-lowering drug for many years [122]. However, after the World Health Organization trial found no reduction in overall cardiovascular events and an increase in overall mortality, the use of clofibrate was declined sharply, in part because of cholecystectomy secondary to death [123]. Many fibric acid analogs have been developed since then. Currently, gemfibrozil and fenofibrate are approved for use in the United States; besides bezafibrate and ciprofibrate are available in Europe [124]. Fenofibrate is an oral prodrug that is converted by esterases into its active metabolite, fenofibric acid [125], which is one of the most widely lipid-lowering agent and usually combines with a statin [126]. Fenofibrate has been used commercially under the brand name Tricor^®^ [127,128] but its use is considerably limited because it has very low bioavailability, chiefly under fasting conditions, due to its poor water solubility and lipophilic nature [129]. Trilipix^®^ (choline fenofibrate, ABT-335) is the newest formulation of a fibric acid derivative approved by the FDA. Trilipix^®^ does not require enzymatic cleavage to become active. Instead, it rapidly dissociates to the active form of free fenofibric acid within the gastrointestinal tract and does not undergo first-pass hepatic metabolism [130]. Fenofibric acid has proven to be safe both as a monotherapy and in combination with statins. In addition, long-term trials have shown that treatment with fenofibric acid combined with statins for up to 2 years in patients with mixed dyslipidemia is safe, in that that no deaths, rhabdomyolysis, or other serious adverse events were reported [126]. The old and well-known lipid-lowering fibric acid derivative bezafibrate is the first clinically tested pan–PPAR activator with a good safety profile [131]. A clinical study, the Bezafibrate Atherosclerosis Coronary Intervention Trial (BECAIT), has shown that the long-term administration of bezafibrate can slow the rate of progression of atherosclerotic lesions in young male post infarction patients and thus reduce the incidence of coronary events [132]. However, from a biochemical point of view, bezafibrate is a PPAR ligand with a relatively low potency. Gemfibrozil, similar to other fibric acid derivatives, has a wide range of potentially favorable effects on lipoprotein metabolism [133]. The VA High-Density Lipoprotein Intervention Trial (VA-HIT), which was conducted with gemfibrozil, is the first lipid intervention trial to show that raising HDL-C concentrations in persons with established coronary heart disease (CHD) and both a low HDL-C and a low LDL-C level will significantly reduce the incidence of major coronary events [116]. Gemfibrozil increases plasma HDL-C by decreasing cholesteryl ester transfer protein-mediated cholesterol exchange from HDL and by directly stimulating hepatic HDL synthesis and secretion [134]. Ciprofibrate is known to decrease TG and TC levels and increase HDL cholesterol levels in hyperlipidemic patients [135]. However, ciprofibrate raises serum creatinine and lowers the activity of hepatic enzymes in the serum [136]. Pemafibrate (K-877) is a novel member of the selective PPARα modulator (SPPARMα) family [137] that was designed to have a higher PPARα agonistic activity and selectivity than existing PPARα agonists (such as fibrates) [138]. Pemafibrate exhibits protective antiatherogenic properties in mice by its TG and remnant lipoprotein-lowering effects, its beneficial effects on HDL metabolism and RCT and its anti-inflammatory activity in macrophages and the arterial wall, resulting in reduced atherosclerosis burden [139]. In phase III clinical trials, compared to fenofibrate, pemafibrate has greater PPARα activation in vitro and lower effects on TGs than fenofibrate. It may become a better choice for patients with metabolic syndrome and T2DM who with residual CV risk [137]. Statins, the favorable agents for lower lipid parameters, combining with fibrates is a better treatment strategy because the two drugs work differently and can complement each other [140,141].The combination of fenofibrate with 20 mg or 40 mg simvastatin was more potent in reducing TG and increasing HDL-C levels than monotherapy with simvastatin or fenofibrate separately [142]. In addition, another drug, pravastatin sodium/fenofibrate, is also on the market for dyslipidemia treatment. However, statin–fibrate combination should be attention due to increasing risk of myopathy and rhabdomyolysis [143].

Forty years after the introduction of the first fibrate in clinical practice, the exact role of these pharmacologic compounds remains ill-defined [144]. Hence, there are still novel PPAR agonists intended for dyslipidemia treatment in the clinical research stage, as shown in Table 9.

Nonalcoholic steatohepatitis (NASH) defines a subgroup of nonalcoholic fatty liver disease where liver steatosis coexists with hepatic cell injury (apoptosis and hepatocyte ballooning) and inflammation [145]. It occurs in close association with obesity, T2DM and cardiometabolic conditions that define the metabolic syndrome [146]. Elafibranor is a selective dual agonist against PPARα/δ that has demonstrated efficacy in disease models of NAFLD/NASH and liver fibrosis [147]. Elafibranor exerts its major effects through the transcriptional regulation of key genes involved in hepatic lipid and glucose metabolism but also modulates hepatic inflammation and collagen turnover [147]. In phase III trials, elafibranor consistently improved plasma lipids and glucose homeostasis, peripheral and hepatic insulin resistance and liver inflammatory markers in dyslipidemic, prediabetic and T2DM patients [148,149]. Three prescription OM3-FAs (eicosapentaenoic acid (EPA) and docosahexaenoic acid (DHA)) have been approved for the management of severe hyperlipidemia [150]. Icosabutate, a first-in-class synthetic, structurally enhanced omega-3 fatty acid derivative, has PPARα activity but with potentially important differences from the fibrates and OM3-FAs. Preclinical observations proved to be consistent with results from an exploratory phase Ib study in hypercholesterolemic subjects, in which icosabutate significantly reduced TGs, ApoC3 and low-density lipoprotein cholesterol (LDL-C) [151]. KD-3010, a dual PPARβ/δ agonist, is under development by Kalypsys. Kalypsys has demonstrated activity in animal models of nonalcoholic steatohepatitis, high fat diet-induced obesity and the ob/ob mouse. Phase Іa safety/tolerability studies have been completed and a phase Іb dose-range study was begun in 2007 [152]. In addition, there are many drugs at the clinical research stage, including ZYH-7 (phase II, Zydus Cadila), CER-002 (phase І, Nippon Chemiphar) and GSK-625019 (phase І, GlaxoSmithKline).

There are also PPAR ligand drugs intended for the treatment of dyslipidemia whose development was terminated in the clinical research stage. We summarize these drugs as follows (Table 10).

The treatment of mixed dyslipidemia is fraught with difficulty because of the need to reduce LDL-C and TG levels while trying to elevate HDL-C levels. For this purpose, combination drug therapy is often the only effective option. Unfortunately, the drug combinations utilized for mixed dyslipidemia potentially increase the risk for adverse events. Rosuvastatin, the newest in its class, is the most potent statin currently available and provides significant reductions in LDL-C and TG and elevations in HDL-C. When used in combination to treat mixed dyslipidemia, rosuvastatin and fenofibrate or rosuvastatin and fenofibric acid demonstrate beneficial effects in this patient population and are well tolerated with no increased risk of adverse events [153]. In addition, many drugs have been terminated at the clinical research stage, including GW-501516 (phase II, GlaxoSmithKline), GFT 14 (phase II, Genfit), GW-544 (phase І, GlaxoSmithKline), DFR-11605 (phase І, Dr Reddys Laboratories), MP-136 (phase І, Mitsubishi Tanabe Pharma), DRF-10945 (phase І, Dr Reddys Laboratories), NS-220 (phase І, Nippon Shinyaku Pharma) and F-16482 (phase І, Pierre Fabre).

### 3.2. Cardiovascular Diseases (CVDs)

Dyslipidemia is one of the major risk factors for CVD and plasma TG levels are a strong predictor of CVD [154]. CVDs are the leading cause of mortality and morbidity, accounting for 31% of all deaths worldwide. Of all deaths due to CVD, approximately 80% are due to CHD or stroke. Numerous studies have shown that blood cholesterol-lowering therapy reduces the occurrence of atherosclerotic cardiovascular disease (ASCVD) [155]. 3-Hydroxy-3-methyl-glutaryl-coenzyme A reductase (HMG-CoA) inhibitors or statins have demonstrated a significant reduction in CVD risk in a large number of landmark trials [156]. However, 70% of risk remains even after the treatment of high LDL-C by statins [157]. To further reduce this risk, fibrates are recommended to manage elevated TG and low HDL-C levels.

Hence, dual therapy of statins with fibrates can improve triglyceride and HDL-C levels more than monotherapy with equivalent dose statins, as shown in Table 11.

Pitavastatin is a competitive inhibitor of HMG-CoA reductase, the enzyme that stimulates the production of mevalonate, which is the rate-determining step in cholesterol biosynthesis [158]. The use of drugs that inhibit this enzyme has been associated with reductions in TC and LDL-C in a dose-dependent manner [159]. The co-administration of fenofibrate with pitavastatin for 7 days was found to be safe, well tolerated and without clinically significant PK interactions [160]. Furthermore, low doses of pitavastatin and fenofibrate were both effective in decreasing sd-LDL-C concentration and reduction [161]. In addition to the co-administration of fibrate with stain, there are other drugs in the clinical research stage. Gemcabene calcium is a small molecule, the monocalcium salt of a dialkyl ether dicarboxylic acid with the chemical name 6,6′-oxybis(2,2-dimethylhexanoic acid) monocalcium salt and is currently in late-stage clinical development. In rodents, gemcabene showed varying targets, including apoC-III, apoA-I and peroxisomal enzymes, which are considered to be regulated via PPAR gene activation, suggesting a PPAR-mediated mechanism of action for the observed hypolipidemic effects observed in rodents and humans [162]. By inhibiting interleukin-1 beta (IL-1β) -induced inflammation and CRP production and resulting in improvements in CVD events through inhibiting IL-1β, canakinumab, in the Canakinumab Anti-inflammatory Thrombosis Outcomes Study (CANTOS) study [163] and gemcabene have shown hypolipidemic and anti-inflammatory properties, in addition to LDL lowering activity, which offers an added benefit to CVD patients [164]. KRP-105, developed by Kyorin, is a highly selective PPARα agonist. In addition to improving the lipid metabolism, KRP-105 increased adiponectin, reduced leptin and suppressed weight gain in animal models, suggesting its potential as a unique antidyslipidemia agent. However, KRP-105 was discontinued from development as part of the company’s R & D strategy [165].

## 4. PPAR Ligand Therapeutics in Other Diseases

PPARs are not only drug targets of glucose and lipid metabolism but also can be used to treat other diseases, such as primary biliary cholangitis, gout, cancer, AD and ulcerative colitis. Here, we summarize the PPAR ligand drugs for the treatment of other diseases in the clinical research stage (Table 12).

Functional studies of PPARδ are still in its infancy and there are increasing evidences that ubiquitously expressed PPARδ has multiple effects and can control a variety of physiological processes, mainly including lipid and lipoprotein metabolism regulation [166,167], insulin sensitivity [168], cardiac function [169], epidermal biology [170], neuroprotection [171] and gastrointestinal tract function and disease [172] Primary biliary cholangitis is a progressive cholangitic liver disease that, if untreated, progresses to cirrhosis and death or liver transplantation [173]. Two types of drugs are currently approved for the medical treatment of primary biliary cholangitis (PBC), ursodeoxycholic acid and obeticholic acid [174] but both have certain adverse effects [174,175]. Seladelpar, a selective PPARδ agonist, is a new therapy for PBC through regulating the cholesterol transporter ABCG5/ABCG8 [176]. Seladelpar appeared safe and well tolerated with no specific adverse reaction definitively associated with the drug [176]. Seladelpar reduces the number of macrophages, fibrosis and other markers of stellate cell activity in a mouse model [177]. In patients with mixed dyslipidemia [176] or homozygous familial hypercholesterolemia, seladelpar reduced LDL-C and induced sustained decreases in biochemical markers of cholestasis such as alkaline phosphatase, γ-glutamyl transpeptidase (GGT) and total bilirubin [178]. In phase Ш trials, seladelpar treatment normalized alkaline phosphatase levels but this treatment was associated with grade 3 increases in aminotransferases and the study was stopped early. Accordingly, the effects of seladelpar at lower doses should be explored.

Gout is the most common cause of inflammatory arthritis and has a major impact on quality of life [179,180]. Chronic hyperuricemia, the biochemical signature of the disease, leads to the deposition of urate crystals in articular structures and the disruption of these crystals is believed to trigger flares [181]. Arhalofenate, a selective partial PPARγ modulator, is a single enantiomer of halofenate and developed as a lipid-lowering agent [182,183]. Recently, arhalofenate was proven to be a uricosuric drug that lowers serum UA by blocking its reabsorption by the inhibition of URAT1 [184] in the proximal tubules of the kidney. Additionally, arhalofenate has been suggested to exert a potent anti-inflammatory effect [184]. In the phase IIb study, arhalofenate at a dosage of 800 mg decreased gout flares significantly compared to allopurinol at a dosage of 300 mg [184]. Another dual PPARα/γ agonist, oxeglitazar, whose development was halted in phase І clinical trials, is also used for gout treatment.

Over the course of several decades of research, evidence has emerged that Alzheimer’s disease (AD) is quite complex and is associated with a multitude of cellular, biochemical and molecular abnormalities [185]. In fact, AD could be regarded as a brain form of diabetes, since insulin resistance and deficiency develop early and progress with the severity of neurodegeneration [186]. T3D-959 is a small-molecule dual agonist of PPARδ/γ [185] and has clear effects that preserve spatial learning and memory in an established experimental model of sporadic AD [186]. In a phase IIa trial, T3D-959 significantly improved motor performance and preserved both cortical and normalized white matter structure via the agonism of PPARδ and PPARγ in AD model rats [186].

Lung cancer is one of the highest cancer deaths worldwide and more than 60% of lung cancer patients are already in an incurable stage of diagnosis [187,188]. For many years, platinum-based doublet chemotherapy has become the most common treatment for patients with advanced non-small cell lung cancer (NSCLC) [189]. However, excessively toxic chemotherapy is also a concern for the public. PPARγ has been shown to possess antitumor properties in preclinical models of human cancers, including NSCLC [190,191]. Efatutazone is a novel third-generation thiazolidinedione that selectively activates PPARγ-mediated transcription with little effect on other PPAR subtypes [192]. Efatutazone is at least 50 times more potent than rosiglitazone and 500 times more potent than troglitazone for PPAR response element activation and the inhibition of cancer cell growth [193]. In a phase I study, efatutazone demonstrated acceptable tolerability with evidence of disease control in patients with advanced malignancies [192]. In addition, efatutazone inhibits the proliferation of human pancreatic and anaplastic thyroid tumor-cell cultures [194]. Daiichi Sankyo (originator of efatutazone hydrochloride) reinitiated enrolment in a phase II trial of efatutazone for the treatment of thyroid cancer. Another agonist of PPARγ, etalocib sodium (LY293111), which is a biphenyl-substituted diaryl ether carboxylic acid, is also a potential agent for the medical treatment of NSCLC [195]. In a phase I study, oral LY293111 was generally well tolerated, with a recommended phase II dose of 600 mg orally twice daily [196]. LY has also been found to inhibit pancreatic cancer cell lines as well as human pancreatic xenografts [197]. The development of LY-293111 for NSCLC treatment has subsequently been discontinued; however, clinical research on its effect on pancreatic and other cancers are ongoing.

Recent epidemiological data show that the incidence and prevalence of ulcerative colitis (UC) are increasing in many parts of the world [198]. PPARγ has been shown to be expressed in macrophages [199], dendritic cells (DCs) [200] and T and B lymphocytes [200]. More importantly, rosiglitazone was shown to be effective in the treatment of mild to moderately active UC [201]. (*R*)-(−)-GED-0507-34 has demonstrated 100- to 150-fold higher PPARγ activation than 5-ASA in vitro using Caco-2 cells transfected with PPRE-Luc reporter system [202]. None of these deleterious events has been observed with the new PPARγ modulator GED-0507-34, even when used at high concentrations during toxicological studies performed in rats, dogs and rabbits and no side effects were observed in the phase I study performed in 24 healthy subjects [202]. This new molecule is currently in phase II of clinical trials [203]. IVA337, the pan-PPAR agonist, is a therapeutic agent for systemic sclerosis through improving inflammatory and fibrosis [204]. There are many drugs used in the treatment of other diseases, including OMS-403 (phase II, Opioid abuse, Smoking cessation), fonadelpar (phase II, Corneal disorders), IVA-337 (phase II, Systemic sclerosis), macuneos (phase І, Age-related macular degeneration), MA-0211 (phase І, Duchenne’s muscular dystrophy).

## 5. Discussion

**Figure 2:**
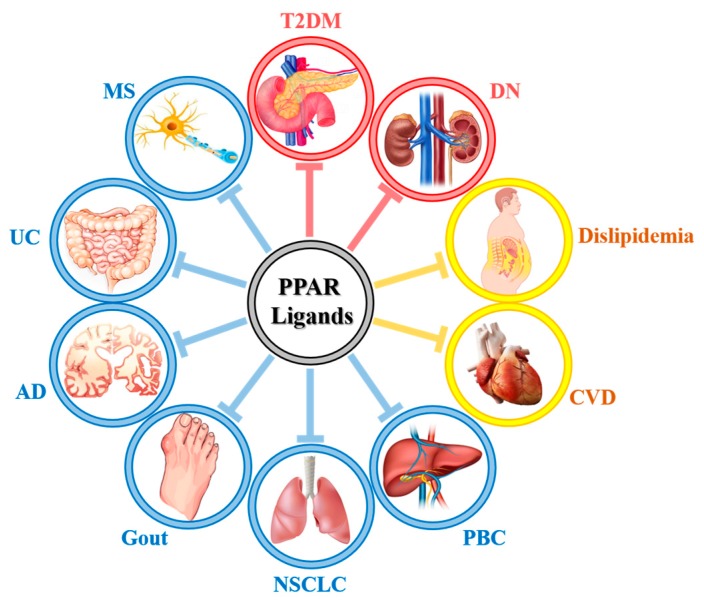
Concept map of the PPAR ligands in various kinds of diseases. T-bar: inhibition.

Metabolic abnormalities, including T2DM, dyslipidemia, NAFLD and CVD, are a worldwide epidemic that seriously endangers global health. Considering the wide range of roles involved in energy homeostasis and cell proliferation/apoptosis, PPAR agonists are suggested for the treatment of metabolic disorders. In this study, we comprehensively summarized the roles of PPAR synthetic ligands in current clinical applications or studies for the treatment of T2DM, DN, obesity, CVDs, MS, AD, gout, cancer, PBC, UC et al., as shown in Figure 2.

Diabetes treatment drugs represented by TZDs, which mainly activate PPARγ, have received widespread attention and are focuses for drug development. Over the past decades, in addition to the eight existing TZD drugs that have been approved and used in clinical treatment, many drugs are still in clinical studies or have even been discontinued. The use of TZDs for diabetes treatment in humans has been limited by side effects, including edema, weight gain and worsening of CHF. Thus, an increasing number of partial PPARγ agonists or SSPARMs, such as INT131 and MK0533, have been developed to reduce the side effects while improving insulin sensitivity. In a recent study, we reported that DBZ (danshensu bingpian zhi), a putative PPARγ agonist, simultaneously prevented HFD-induced obesity-related metabolic syndrome and gut dysbiosis. It also has antiatherosclerotic effects that involve inflammation suppression and the promotion of reverse cholesterol transport through concurrent partial activation of both PPARγ and LXRs [4,205,206]. Drugs for treating dyslipidemia via activating PPARα, especially represented by fibrates, are also widely used. Fibrate decreases the level of triglyceride-rich lipoproteins in serum by increasing the gene expression involved in fatty acid-β-oxidation and a decrease in apolipoprotein C-III gene expression [207]. Furthermore, PPARα agonists can increase the stability of atherosclerotic plaques and reduce the accumulation of hepatic fat accumulation, leading the party to NASH/NAFLD and reducing the risk of CVD. PPARα agonists have few adverse effects but do generally increase the plasma levels of homocysteine and creatinine, which must also be emphasized [208]. PPARδ is ubiquitously expressed and a target for management by the different components of metabolic syndrome. Clinical trials on selected PPARδ agonists have assessed both metabolic and vascular outcomes and no severe side effects have been reported to date, except for GW1516, which induced cancer in several organs in rodents [209]. Any differential mechanism of PPARδ action in different tissues should be explored in order to develop new PPARδ agonists with improved efficacy and safety. In addition to modulating lipid and glucose metabolism, PPAR agonists play significant roles in several diseases, including primary biliary cholangitis, gout, AD, non-small cell lung cancer and UC.

Currently used agonists are still at a relatively preliminary stage, the potency is weak (as is the case for PPARα), or there are many side effects (such as in PPARγ). In the past decade, increasing numbers of compounds have been developed, including dual PPAR agonists (PPARα/γ, PPARα/δ and PPARδ/γ) and pan-PPAR agonists or selective modulators. For example, clofibric acid and fenofibric acid are dual activators of PPARα and PPARγ, with a selectivity to PPARγ of about 10-fold. In addition, bezafibrate, another fibric acid that activates all three PPAR subtypes (α, γ and δ), has a broader role [131]. Unfortunately, the development of diverse dual PPAR agonists has not met with the anticipated success. Their development has thus far been halted in late-phase clinical trials because of reported side effects, such as increased cardiovascular risk (muraglitazar), carcinogenicity (ragaglitazar and MK-767), liver toxicity (imiglitazar) and renal injury (tesaglitazar) [210]. In this article, we summarize the current PPAR ligands in clinical drug discovery and development. We hope that more powerful dual PPAR agonists or pan-PPAR agonists will be highly effective in a clinical setting of patients with coexisting relevant lipid and glucose metabolism disorders.

## Figures and Tables

**Table 1:** Medications of PPAR synthetic ligands in currently clinical applications or studies.

Indication	Development Status				Total
	In Market	Withdrawn	Clinical Research	Discontinued in Clinical Research	
Type 2 diabetes	3	1	5	23	32
Diabetic diseases	1	0	5	10	16
Dyslipidemia	7	0	6	8	21
CVDs	0	0	1	1	2
Other diseases	0	0	12	1	13

**Table 2:** Approved drugs of PPAR ligands for type 2 diabetes treatment.

Generic Name	Type of PPAR Agonist	Molecular Weight	Company
Rosiglitazone Maleate	PPARγ agonist	473.5	GlaxoSmithKline
Pioglitazone Hydrochloride	PPARγ agonist	392.898	Takeda(Originator) Lilly
Lobeglitazone Sulfate	Dual PPARα/γ agonist	578.61	Chong Kun Dang

**Table 3:** Drugs of PPAR ligands for type 2 diabetes treatment in clinical stage.

Generic Name	Type of PPAR Agonist	Molecular Weight	Company	Development Status
Chiglitazar	PPARs agonist	594.61	ChipScreen	Phase Ш active
KDT-501	PPARα agonists	404.588	KinDex Pharmaceuticals	Phase II active
Naveglitazar	PPAR modulator	422.477	Lilly(Originator)Ligand (Originator)	Phase II Pending
AVE-0897	Dual PPARα/γ agonist	469	Genfit(Originator)Sanofi	Phase І active
ZY-H2	Dual PPARα/γ agonist	unknown	Zydus cadila	Phase І Pending

**Table 4:** Dual PPARα/γ agonist “glitazar” for type 2 diabetes treatment.

Generic Name	Type of PPAR Agonist	Molecular Weight	Company	Development Status
Aleglitazar	Dual PPARα/γ agonist	437.51	Roche	Phase Ш discontinued
Ragaglitazar	Dual PPARα/γ agonist	419.477	Novo Nordisk Pharmaceutical	Phase Ш discontinued
Imiglitazar	Dual PPARα/γ agonist	470.525	Takeda	Phase Ш discontinued
Tesaglitazar	Dual PPARα/γ agonist	408.465	AstraZeneca	Phase Ш discontinued
Peliglitazar	Dual PPARα/γ agonist	530.577	Bristol-Myers Squibb	Phase II discontinued
Farglitazar	Dual PPARα/γ agonist	546.623	GlaxoSmithKline	Phase II discontinued
Sipoglitazar	Dual PPARα/γ agonist; Insulin sensitizer	463.552	Takeda	Phase II discontinued
Reglitazar	Dual PPARα/γ agonist	392.411	Japan Tbacco(Originator) Pfizer	Phase II discontinued
Indeglitazar	Dual PPARα/γ agonist	389.422	Pfizer	Phase II discontinued
Muraglitazar	Dual PPARα/γ agonist	516.55	Bristol-Myers Squibb	NDA Filing US

**Table 5:** Drugs of PPAR ligands for treatment of type 2 diabetes discontinued in clinical stage.

Generic Name	Type of PPAR Agonist	Molecular Weight	Company	Development Status
Troglitazone	PPARγ agonists	441.542	Daiichi Sankyo (Originator) Pfizer	Withdrawn
Rivoglitazone Hydrochloride	PPARγ agonists	433.907	Daiichi Sankyo (Originator) Santen	Phase Ш discontinued
Balaglitazone	Partial agonist of PPARγ	395.433	Dr Reddy’s Laboratories (Originator) Rheoscience	Phase II discontinued
FK-614	PPARγ agonists; Insulin sensitizer	468.393	Astellas (Originator) Aestus Therapeutics	Phase II discontinued
Ciglitazone	PPAR agonists	333.446	Takeda	Phase II discontinued
ONO 5129	Dual PPARα/γ agonist	unknown	Ono	Phase II discontinued
EML-4156	Dual PPARα/γ agonist	314.381	Merck Serono	Phase II discontinued
Netoglitazone; Isaglitazone	Dual PPARα/γ agonist	381.421	Mitsubishi Tanabe Pharma (Originator) Perlegen Sciences	Phase II discontinued
PN-2034	PPARγ agonist	unknown	Wellstat (Originator) Sanofi	Phase II discontinued
Edaglitazone	PPARγ agonists	464.554	Roche	Phase II discontinued
Darglitazone Sodium	Dual PPARα/γ agonist	442.465	Pfizer	Phase І discontinued
AVE-5376	Dual PPARα/γ agonist	unknown	Sanofi (Originator)	Phase І discontinued
DS-6930	PPARγ agonists	136.129	Daiichi Sankyo	Phase І discontinued
E-3030	Dual PPARα/γ agonist	481.93	Eisai	Phase І discontinued

**Table 6:** Drugs of PPAR ligands for treatment of diabetic associated complications in market or clinical stage.

Generic Name	Type of PPAR Agonist	Indication	Molecular Weight	Company	Development Status
Saroglitazar	Dual PPARα/γ agonist	Diabetic dyslipidemia	439.57	Zydus cadila	Approved
AMG-131	PPARγ agonist	Type 2 diabetes; Multiple sclerosis (MS)	672.38	Amgen (Originator) InteKrin Therapeutics	Phase II active
K-111	PPARα agonists	Type 2 diabetes; Hyperlipidemia	379.75	Roche	Phase II Pending
CLX-0921	PPARγ agonist	Type 2 diabetes; Rheumatoid arthritis (RA)	519.568	Theracos	Phase II Pending
HPP 593	PPARδ	Diabetes Dyslipidemia	unknown	vTv Therapeutics LLC	Phase II active
SAR-351034	PPAR agonists	Type 2 diabetes; Dyslipidemia	unknown	Sanofi	Phase І active

**Table 7:** Drugs of PPAR ligands for treatment of diabetic associated complications terminated in clinical stage.

Generic Name	Type of PPAR Agonist	Indication	Molecular Weight	Company	Development Status
MK-0767	Dual PPARα/γ agonist	Type 2 diabetes; Dyslipidemia	422.36	Kyorin (Originator) Merck Sharp & Dohme	Phase Ш discontinued
Cevoglitazar	Dual PPARα/γ agonist	Type 2 diabetes; Lipodystrophy	558.528	Novartis	Phase II discontinued
Sodelglitazar	Pan–PPAR agonists; Insulin sensitizer	Type 2 diabetes; Hyperlipidemia	499.539	GlaxoSmithKline	Phase II discontinued
AVE-0847	Dual PPARα/γ agonist	Type 2 diabetes; Lipodystrophy	unknown	Sanofi	Phase II discontinued
KRP-101	PPARα agonists	Diabetes; Dyslipidemia	451.49	Kyorin	Phase II discontinued
DSP-8658	Dual PPARα/γ agonist	Type 2 diabetes; Alzheimer’s disease	unknown	Dainippon Sumitomo	Phase І discontinued
ARH-049020	PPAR agonists	Type 2 diabetes; Insulin resistance	429.51	AstraZeneca	Phase I discontinued
LY-510929	Dual PPARα/γ agonist	Type 2 diabetes; Hyperlipidemia	463.55	Lilly	Phase I discontinued
GSK-376501	PPARγ agonist	Type 2 diabetes; Hypercholesterolemia	531.649	GlaxoSmithKline	Phase I discontinued
Tetradecylthioacetic acid	Pan–PPAR agonists; Lipid Peroxidation inhibitors	Type 2 diabetes; Dyslipidemia	288.49	Badische Anilin-und-Soda-Fabrik	Phase I discontinued

**Table 8:** Drugs of PPAR ligands for treatment of dyslipidemia in market.

Generic Name	Type of PPAR Agonist	Indication	Molecular Weight	Company
Clofibrate	PPAR agonists	Hyperlipidemia Hypertriglyceridemia Hypercholesterolemia	242.699	Pfizer
Fenofibrate; Fenomax	PPARα agonists	Hypercholesterolemia Hypertriglyceridemia	360.834	Abbvie
Choline Fenofibrate	PPARα agonists	Hyperlipidemia	421.918	Abbvie
Bezafibrate	Pan–PPAR agonists	Hypertriglyceridemia Hypercholesterolemia Mixed hyperlipidemia	361.822	Unknown
Gemfibrozil	PPAR agonists	Hyperlipidemia; Ischemic heart disorder	250.338	Pfizer
Ciprofibrate	PPAR agonists	Hyperlipidemia	289.152	Unknown
Pemafibrate	PPARα agonists	Dyslipidemia	490.556	Kowa

**Table 9:** Drugs of PPAR ligands for treatment of dyslipidemia in clinical stage.

Generic Name	Type of PPAR Agonist	Indication	Molecular Weight	Company	Development Status
Elafibranor	Dual PPARα/δ agonist	Non-alcoholic fatty liver disease (NAFLD); Dyslipidemia; Type 2 diabetes	384.49	Genfit	Phase III active
Icosabutate	PPAR agonists; Cholesterol ester transfer protein inhibitors	Hypertriglyceridemia	374.565	BASF	Phase II active
ZYH-7	PPARα agonists	Dyslipidemia	unknown	Zydus cadila	Phase II active
CER-002	PPARδ agonists	Dyslipidemia	unknown	Nippon Chemiphar	Phase І active
GSK-625019	PPAR agonists	Metabolic Syndrome X; Type 2 diabetes	unknown	GlaxoSmithKline	Phase І Pending
KD-3010	PPARα agonists	Obesity; Diabetes; Dyslipidemia	670.72	Kalypsys	Phase І Pending

**Table 10:** Drugs of PPAR ligands for treatment of dyslipidemia discontinued in clinical stage.

Generic Name	Type of PPAR Agonist	Indication	Molecular Weight	Company	Development Status
GW-501516	PPARδ agonists	Hyperlipidemia	453.494	GlaxoSmithKline	Phase II discontinued
GFT 14	PPARα agonists	Dyslipidemia	unknown	Genfit	Phase II discontinued
GW-544	Dual PPARα/γ agonist	Hyperlipidemia	510.58	GlaxoSmithKline (Originator)Ligand	Phase І discontinued
DFR-11605	PPAR agonists	Obesity	unknown	Dr Reddys Laboratories (Originator)Perlecan	Phase І discontinued
MP-136	PPARα agonists	Dyslipidemia	unknown	Mitsubishi Tanabe Pharma	Phase І discontinued
DRF-10945	PPARα agonists	Lipid metabolism disorders	unknown	Dr Reddys Laboratories (Originator)Perlecan	Phase І discontinued
NS-220	PPARα agonists	Lipid metabolism disorders	373.449	Nippon Shinyaku Pharma	Phase І discontinued
F-16482	PPAR modulator	Metabolic Syndrome X	unknown	PIERRE FABRE	Phase І discontinued

**Table 11:** Drugs of PPAR ligands for treatment of cardiovascular disease (CVD).

Generic Name	Type of PPAR Agonist	Indication	Molecular Weight	Company	Development Status
Gemcabene Calcium	PPAR agonists	Hypercholesterolemia	340.473	Gemphire Therapeutics	Phase II active
KRP-105	PPARα agonists	Hypercholesterolemia	unknown	Kyorin	Phase І discontinued

**Table 12:** Drugs of PPAR ligands for treatment of other diseases in clinical stage.

Generic Name	Type of PPAR Agonist	Indication	Molecular Weight	Company	Development Status
Seladelpar lysine dihydrate	PPARδ agonists	Primary biliary cirrhosis	626.685	Janssen (Originator) CymaBay Therapeutics	Phase Ш active
Arhalofenate	Partial PPARγ modulators	Chronic gout	415.793	CymaBay Therapeutics	Phase II active
T3D-959	Dual agonist of PPARδ/γ	Alzheimer’s disease	443.47	DARA BioSciences	Phase II active
Efatutazone hydrochloride	Selectively activates PPARγ	Thyroid cancer; Non-small cell lung cancer; Colorectal cancer	593.52	Daiichi Sankyo	Phase II Pending
IVA-337	PPAR agonists	Systemic sclerosis	434.92	Abbvie(Originator)Inventiva	Phase II active
Fonadelpar	PPAR agonists	Corneal disorders	504.524	Senju Pharmaceuticals	Phase II active
OMS-403	PPARγ agonists	Opioid abuse Smoking cessation	unknown	Omeros	Phase II active
10-Nitrooctadec-9-enoic acid	PPARγ ligands; Transcription factor modulators; Inflammation mediator modulators	Acute kidney injury Renal failure	327.465	Complexa	Phase І active
GED-0507-34	PPAR modulator	Inflammatory bowel disease	unknown	Giuliani	Phase І active
Macuneos	PPARα agonists	Age-related macular degeneration	unknown	Biophytis	Phase І active
MA-0211	PPARδ modulators	Duchenne muscular dystrophy	unknown	Astellas	Phase І active
Oxeglitazar	Dual PPARα/γ agonist	Gout	314.381	Merck Serono	Phase І Pending
Etalocib sodium	PPARγ agonists; 5-Lipoxygenase inhibitor; Leukotriene B4 receptor antagonist	Pancreatic cancer; Non-small cell lung cancer	566.601	Lilly(Originator)Vernalis	Phase II discontinued
